# Abortion beyond a medical issue; women's perception on the current Ethiopian abortion law among reproductive‐aged women by 2023, a community‐based cross‐sectional study

**DOI:** 10.1002/hsr2.2314

**Published:** 2024-08-12

**Authors:** Besfat Berihun Erega, Addisu Molla, Hiwot Dejen, Wassie Yazie Ferede

**Affiliations:** ^1^ Department of Midwifery, College of Health Sciences Debre Tabor University Debre Tabor Ethiopia

**Keywords:** abortion law, Ethiopia, perception, women

## Abstract

**Background:**

A nationwide study on the contribution of abortion to maternal mortality in Ethiopia was 6%−9%. To bring Ethiopia's legal system into compliance with the country's new constitution, a new criminal code was created in 2005. In the new penal code, after 2005, abortion was permitted under broad circumstances; in the case of rape, incest, or fetal impairment; if pregnancy continuation or birth would endanger the health or life of the woman or fetus; if the woman has physical or mental disabilities; and if the woman is a minor who is physically or mentally unprepared for childbirth.

**Objectives:**

To determine the status of women's perception toward the current Ethiopian abortion law among reproductive‐aged women.

**Method:**

A community‐based study was conducted from June 10 to October 30, 2023. Chi‐square test and multivariable logistic regression methods were employed using SPSS 23. The strength of associations and the significance level was examined using *p*‐value and odds ratio at 95% CI, respectively.

**Result:**

The prevalence of women's positive perception toward Ethiopian abortion law among reproductive‐aged women is 21.18%. The age group of between 18 and 24, educational status of being unable to read and write, living solely, history of unplanned pregnancy, and age of marriage under 18 were associated with women's perception to the current abortion law of Ethiopia.

**Conclusion:**

The finding of this study is unexpectedly low, which needs exhaustive intervention.

## 1 INTRODUCTION

Worldwide, more than 47,000 women died and millions face major injuries due to abortion complications every year.^1^, ^2^ While access to safe abortion services is restricted in many parts of sub‐Saharan Africa, the government of Ethiopia is trying to address abortion‐related maternal morbidity and mortality by preparing partial allowance for safe abortion.^3^, ^4^ Before 2005 the contribution of unsafe abortion to maternal mortality had been 32%, changing the law to improve access to safe abortion service.^5^

The World Health Organization (WHO) states that an enabling regulatory and policy environment is needed to ensure that every legally eligible woman has ready access to safe abortion care.^6^, ^7^, ^8^ Abortion was legally restricted in almost every country by the end of the nineteenth century. The most important sources of such laws were the imperial countries of Europe—Britain, France, Portugal, Spain, and Italy—who imposed their laws forbidding abortion on their colonies.^9^, ^10^

In Africa, only a few North African countries have signed or ratified the Maputo Protocol, and those few have done so only very recently. In 2015, Beji Essebsi, then president of Tunisia, signed the protocol, but Tunisia did not ratify it until 2018, and laws have yet to be harmonized according to its principles. Sudan has signed but not ratified it.^11^, ^12^, ^13^

A nationwide study on the contribution of abortion to maternal mortality in Ethiopia was 6%−9%.^5^, ^14^ To bring Ethiopia's legal system into compliance with the country's new Constitution, a new criminal code was created in 2005.^3^, ^5^ Abortion remained illegal after a period of fervent agitation and discussion, but some notable exceptions were created that permitted the growth and integration of services within the public health system. Determining the degree of service implementation required both the passing of the legislation and the creation of technical guidelines.^5^, ^15^, ^16^

In addition, abortion was allowed only to save the life of the women in the previous criminal code. In the new penal code, after 2005, abortion was permitted under broad circumstances; in the case of rape, incest, or fetal impairment; if pregnancy continuation or birth would endanger the health or life of the woman or fetus; if the woman has physical or mental disabilities; and if the woman is a minor who is physically or mentally unprepared for childbirth.^15^, ^16^, ^17^, ^18^

Available studies on different parts of Ethiopia revealed the maternal level of education, paternal level of education, age, marital status, religion, educational status, history of induced abortion, knowledge of legalization of abortion, preference of abortion if the pregnancy is unwanted, and knowledge about the complication of abortion is associated with attitude toward the current abortion law of Ethiopia.^19^, ^20^, ^21^, ^22^, ^23^, ^24^, ^25^, ^26^

Ethiopia is still in transition from unsafe abortion to safe abortion, having changed its national abortion penal code in 2005. Thus, studying women's perception of the legal provision of safe abortion services is vital. Abortion is more than a medical issue, it has ethical, legal, and human issues involving women and men as individuals and as members of a community. Despite these limited studies being conducted about the acceptability of women on the current abortion law, even available studies focused on educated groups who are known to have better attitudes.^16^, ^27^ Therefore, our study aimed to reveal the perceptions of women on the current Ethiopian abortion law in the general population.

### 1.1 Objective

To determine women's perceptions toward the current Ethiopian abortion law among reproductive‐aged women.

## 2 METHODS AND MATERIALS

### 2.1 Study design

A community‐based cross‐sectional study was employed.

### 2.2 Study setting

The study was conducted at Debre Tabor town in North‐west Ethiopia from June 10 to October 30, 2023. Debre Tabor town is the seat of the south Gondar zone and is administratively divided into three sub‐cities, namely Tewodros, Taytu, and Gebrye.

### 2.3 Sample size determination and sampling technique

The sample size was determined using a previous study done^20^ with 38% proportion of women having a good attitude to abortion law (*p* = 0.38) (level of significance of the population was taken to be 95%, *Z α*/2 = 1.96) and a 5% level of precision (*d* = 0.05). Finally, the sample size became 369, adding 10% nonresponse rate it became 406. A quota sampling technique was carried out until the required number of cases was reached.

### 2.4 Data collection and analysis procedures

Data were collected by six BSc midwives after 2 days of training was given. The data were collected at a household level using an Amharic version interviewer administered a structured questionnaire. The tool was initially prepared in English and translated into Amharic. The tools were piloted for suitability, validity, and reliability for the current study. SPSS software version 16 was used for the analysis. After the data collection, it was checked for its completeness and internal consistency and analyzed using descriptive statistics. Crude odds ratio (COR) and adjusted odds ratio (AOR) with a 95% confidence interval from bivariate and multivariate analyses were used to measure the association between dependent and outcome variables. To see the model fitness, the Hosmer−Lemeshow Goodness of Fit test and multicollinearity were checked to minimize bias. The final model's goodness of fit was assessed using the Hosmer–Lemeshow Goodness of Fit Test, with *p*‐values greater than 0.05 considered as the model's fit to the logistic regression.^16^

### 2.5 Quality control measures

All data collection instruments were pretested at Nefas Mewcha town. Five percent of sample size was employed for pretest and revision to the data collection instrument was subsequently made. The entire data collection process was also supervised.

### 2.6 Operational definitions

When a woman answered “yes” for more than half of the perception questions, it was deemed that she had a positive perception of the present abortion law in Ethiopia. If not, she was regarded as having a negative perception.^20^

## 3 RESULTS

### 3.1 Distribution of sociodemographic characteristics

A total of 406 respondents were included in this study with a response rate of 100%. The majority of the respondents were in the age group of 25−34 years, Orthodox Christian in religion, and had monthly income greater than 2000ETB (Table 1).

**Table 1:** Distribution of sociodemographic characteristics of the respondents (*N* = 406).

Characteristics	Number	Percent
Age group		
18−24	131	32.25
25−34	194	47.78
≥35	81	19.95
Level of educational		
Unable to read and write	79	17.24
Able to read and write	84	20.69
Primary	121	29.80
Secondary	79	19.46
Higher	43	10.59
Monthly income (ETB)		
≤2000	119	29.31
>2000	287	70.69
Marital status		
Married	247	60.84
Never married	159	31.16
Religion		
Orthodox Christian	288	70.91
Muslim	101	24.87
Protestant	17	4.19
Age at marriage (*n* = 247)		
Less than 18	52	7.15
≥18	195	92.85
Family size		
≤Three	116	29.57
>Four	290	71.43

Abbreviation: ETB, Ethiopian Birr.

### 3.2 Distribution of reproductive and clinical characteristics

The majority of the respondents had previous pregnancies and had no history of induced abortion, unplanned pregnancy, and extramarital pregnancy (Table 2).

**Table 2:** Reproductive and obstetric characteristics of respondents (*N* = 406).

Variables	Category	Number	Percent
Ever been pregnant	Yes	326	80.30
	No	80	19.70
Age at first pregnancy, in years (*n* = 326)	<18	45	13.80
	≥18	281	86.20
Ever had an extramarital pregnancy	Yes	27	6.65
	No	379	93.35
Ever had an unplanned pregnancy	Yes	93	22.90
	No	313	77.10
Ever had an induced abortion	Yes	12	2.96
	No	394	97.04
Ever visited a health institution for abortion services	Yes	57	14.04
	No	349	85.96
Ever used contraceptives	Yes	201	49.51
	No	205	50.49
Ever had family or friend who had an induced abortion	Yes	17	4.18
	No	389	98.02
Ever used a traditional healer for pregnancy termination	Yes	09	2.22
	No	397	97.78

### 3.3 Perception of respondents toward Ethiopian abortion law

The majority of our respondents did not accept the current penal code of abortion and thought that women did not have the right to terminate their pregnancy in any context (Table 3).

**Table 3:** Perception of respondents toward the current abortion law of Ethiopia (*N* = 406).

Characteristics	Category	Number	Percent
Do you accept the Ethiopian legal code of abortion	Yes	76	18.72
	No	330	81.28
Do you believe women had the right to terminate pregnancy	Yes	69	19.99
	No	337	83.01
Do you believe pregnancy from rape should be terminated	Yes	94	23.15
	No	312	76.85
Do you think pregnancy from relatives should be terminated	Yes	152	37.44
	No	254	62.56
Do you believe pregnancy endangering women's life should be terminated	Yes	251	61.82
	No	155	38.18
Do you believe pregnancy before 18 years should be terminated	Yes	45	11.08
	No	361	88.92
Do you believe an anomalous fetus should be terminated	Yes	207	50.99
	No	199	49.01
Do you think a mentally ill women can terminate her pregnancy	Yes	86	21.18
	No	320	78.82

### 3.4 Factors associated with maternal perception of Ethiopian abortion law

In this study, women in an age group of between 18 and 24, having an educational status of unable to read and write, living alone, having a history of unplanned pregnancy, and being married under 18 were associated with women's perception to the current abortion law of Ethiopia (Table 4).

**Table 4:** Factors associated with maternal perception toward the current Ethiopian abortion law (*N* = 406).

Variable	Women's perception to current Ethiopian abortion law			
	Positive (*n* = 86)	Negative (*n* = 320)	COR* (95% CI)	AOR* (95% CI)
	Number (%)	Number (%)		
Age group				
18−24	39 (9.60)	92 (22.66)	5.30 (2.05−6.12)	**3.12 (2.35−5.81)** **
25−34	41 (10.10)	149 (36.70)	3.44 (2.98v8.32)	1.88 (0.37−4.71)
≥35	6 (1.48)	75 (18.47)	1	1
Level of educational				
Unable to read and write	7 (1.72)	72 (17.73)	0.08 (0.04−0.74)	**0.05 (0.01−0.69)** **
Able to read and write	8 (1.97)	76 (18.72)	0.09 (0.01−0.92)	0.07 (0.04−1.08)
Primary	37 (9.11)	84 (20.67)	0.38 (0.21−0.87)	0.22 (0.17−2.95)
Secondary	11 (2.71)	68 (16.75)	0.14 (0.09−0.76)	0.06 (0.02−2.31)
Higher	23 (5.66)	20 (4.93)	1	1
Marital status				
Married	27 (6.65)	220 (54.19)	1	1
Never married	59 (14.53)	100 (24.63)	4.81 (2.89−5.61)	**2.87 (1.85−4.03)** **
Monthly income (ETB)				
≤2000	38 (9.36)	81 (19.95)	2.34 (1.78−5.78)	1.60 (0.22−3.91)
>2000	48 (11.82)	239 (58.87)	1	1
History of unplanned pregnancy				
Yes	22 (5.42)	71 (17.49)	1.21 (1.17−4.07)	**1.15 (1.01−4.70)** ***
No	64 (15.76)	249 (61.33)	1	1
Age at marriage (*n* = 247)				
Less than 18	21 (8.50)	31 (12.55)	1.35 (1.06−4.51)	**1.11 (1.07−3.16)** **
≥18	65 (26.31)	130 (52.63)	1	1

*Note*: Bold values are statistically significant.

Abbreviations: AOR, adjusted odds ratio; COR, crude odds ratio.

** *p* ≤ 0.01

*** *p* ≤ 0.001.

## 4 DISCUSSION

### 4.1 Key findings

The primary finding of this study is determining the status of women's perception and its determinants toward the current Ethiopian abortion law among reproductive‐aged women.

### 4.2 Interpretation

The prevalence of good perception toward Ethiopian abortion law among reproductive‐aged women in Debre Tabor town is 21.18% CI (16.23−25.04). This study finding is lower than studies done in Arba Minch, Mizan Aman, Dire Dawa, Ethiopia, and Armenia.^15^, ^24^, ^25^, ^26^ The possible justification for the lower prevalence could be due to the difference in study area, the community‐based nature of our study design, and difference in time frame. The above‐mentioned studies are institutional based so the perception toward the semi‐liberal penal core could differ. The other possible justification for the lower prevalence might be due to the difference in study population, meaning most of the above‐mentioned studies were done among educated groups like students.^15^, ^25^, ^26^

Concerning the factors affecting women's perception to the current Ethiopia abortion law among reproductive‐aged women, the age group of women between 18 and 24, 3.12 (2.35−5.81), increases the odds of good perception by 3.12 times than those aged more than 35 years. This study finding is different from study done at BahirDar city, Ethiopia where age group of women between 25 and 29 increases the odds of good perception to Ethiopian abortion penal code.^20^ The possible reason for this difference could be the difference in study setting and the changing sexual behavior and reproductive attitude of women over time. The possible justification for the association could be the lower chance of younger women to be affected by cultural taboo, higher reproductive and sexual health awareness, and high likely of being single and having unplanned pregnancies so as induced abortion among younger women than older women.

Educational status of “unable to read and write” 0.05 (0.01−0.69) decreases the odds of a positive (good) perception of the Ethiopian legal code of Ethiopia by 95%. This study finding is in opposition with study done at BahirDar city, Ethiopia where educational status of college and above decreases the odds of perception by 71%.^20^ The difference between the two studies could be explained in terms of the higher‐level abortion‐related taboos in Debre Tabor town, Ethiopia, and their information about the improper implementation of the legal code. The possible explanation for this association could be limited awareness about the legal code and the impact of abortion‐related taboo, considering any form of abortion as an evil act.

Never being married 2.87 (1.85−4.03) increases the odds of a positive perception of Ethiopian abortion law by 2.87 times than being married. This study finding is supported by studies done at Arba Minch and Mizan Aman, Ethiopia.^15^, ^24^ The possible explanation for the association could be due to the higher chance of singles to be younger in age so that younger women will be more informed about reproductive health issues, including abortion.

Having a history of unplanned pregnancy 1.15 (1.01−4.70) also increases the odds of positive perception toward the Ethiopian penal code of abortion by 15% than those who had not. The possible reason for this association could be due to the logical concept that women with unplanned pregnancies will mostly require safe abortion services. Since safe abortion in Ethiopia is under restriction, they can easily understand the logical background of the law because they face the challenge.

Age of marriage of under 18 years 1.11 (1.07−3.16) also decreases the odds of positive perception to the current Ethiopian legal code of abortion by 11% than those of over 18. Although we cannot get a study regarding this finding, the possible justification for the association could be due to the thinking of these girls by considering the scenario under their shoes. But there is no comparative study to be discussed about the association.

## 5 CONCLUSION

The overall prevalence of women's positive perception toward Ethiopian abortion law among reproductive‐aged women is 21.18%, which is too low. The age group of between 18 and 24, educational status of unable to read and write, living solely, history of unplanned pregnancy, and age of marriage under 18 were associated with women's perception to the current abortion law of Ethiopia.

## 6 LIMITATIONS OF THE STUDY

Despite its novelty, the cross‐sectional nature and quota sampling technique employed might reduce the cause‐and‐effect association and generalizability of the study. In addition, due to the urban nature of the study area, it might be difficult to make a conclusion for reproductive‐aged women in the rural area of the country.

## 7 RECOMMENDATION

Ethiopia, as a country with a semi‐liberal abortion legal code, needs to draft a strategy for changing women's awareness about the ethical and legal background of its abortion law.

## AUTHOR CONTRIBUTIONS

Besfat Berihun Erega is the primary author, participated in the conceptualization, design, analysis, and interpretation of the data and drafted the manuscript. All other authors contributed equally in contributed for the design, analysis, and interpretation of the data and critically revised the manuscript for important intellectual content. All authors read and approved the final manuscript. All authors have read and approved the final version of the manuscript and had full access to all of the data in this study and takes complete responsibility for the integrity of the data and the accuracy of the data analysis.

## CONFLICT OF INTEREST STATEMENT

The authors declare no conflict of interest.

## ETHICS STATEMENT

Ethical clearance was obtained from the college health science ethical review board of Debre Tabor University with reference number DTU/1099/23. Verbal informed consent was received from each study participant. Those who are unwilling to participate in the study was omitted. Names and other identifying information were not included in the study. Study participants were told that they have the full right not to participate or withdraw from the study at any time. You have also the right not to answer some of the questions that you do not need to respond. Consent was taken from all subjects for the publication of this manuscript.

## TRANSPARENCY STATEMENT

The lead author, Besfat Berihun Erega, affirms that this manuscript is an honest, accurate, and transparent account of the study being reported, that no important aspects of the study have been omitted and that any discrepancies from the study as planned (and, if relevant, registered) have been explained.

## Data Availability

All data included in this manuscript can be accessed from the corresponding author upon reasonable request through the email address.
